# A gene regulation network controlled by Celf1 protein–*rbpj* mRNA interaction in Xenopus somite segmentation

**DOI:** 10.1242/bio.20135629

**Published:** 2013-08-30

**Authors:** Marie Cibois, Carole Gautier-Courteille, Laurent Kodjabachian, Luc Paillard

**Affiliations:** 1 Université de Rennes 1, Université Européenne de Bretagne, Biosit, CS 34317, 35043 Rennes Cedex, France; 2 CNRS UMR 6290 Institut de Génétique et Développement de Rennes, CS 34317, 35043 Rennes Cedex, France; 3 Institut de Biologie du Développement de Marseille, Aix-Marseille Université, CNRS UMR7288, Case 907, 13288 Marseille Cedex 09, France

**Keywords:** RNA-binding protein, Notch, FGF, retinoic acid, Cyp26a

## Abstract

Somite segmentation is impaired in Xenopus *celf1* morphant embryos. The Celf1 RNA-binding protein targets bound mRNAs for rapid degradation, and antisense approaches demonstrated that segmentation defects in *celf1* morphants were due to a derepression of *rbpj* mRNA. Rbpj protein is a key player of Notch signalling. Because segmentation involves complex cross-talk between several signalling pathways, we analysed how *rbpj* derepression impacted these pathways. We found that *rbpj* derepression stimulated the Notch pathway. Notch positively controlled the expression of *cyp26a*, which encodes a retinoic acid (RA)-degrading enzyme. Thus, *rbpj* derepression led to *cyp26a* overexpression and RA attenuation. It also repressed *fgf8*, consistent with an inhibition of FGF signalling. Pharmacological inhibition of the FGF pathway repressed *cyp26a*, but *rbpj* derepression was sufficient to restore *cyp26a* expression. Hence, while it was known that the FGF pathway antagonized RA signalling through expression of *cyp26a*, our results suggest that Rbpj mediates this antagonism. Furthermore, they show that the post-transcriptional repression exerted by Celf1 on *rbpj* mRNA is required to keep *cyp26a* expression under the control of FGF signalling. We conclude that *rbpj* repression by Celf1 is important to couple the FGF and RA pathways in Xenopus segmentation.

## Introduction

In vertebrates, somites are arranged along the anteroposterior axis of the embryo in an organisation known as segmentation. Somite segmentation is a blueprint for vertebral segmentation in adults, and vertebral disorders, such as congenital scoliosis, may arise from defective somite segmentation (Pourquié, 2011). Segmentation results from the periodic emergence of somites from the presomitic mesoderm (PSM). It depends on cross-talk between a clock and a determination front (Mara and Holley, 2007; Dequéant and Pourquié, 2008; Aulehla and Pourquié, 2010; Gibb et al., 2010). The oscillatory expression, in the posterior PSM, of tens of genes encoding factors of the Notch, FGF, and Wnt signalling pathways supports the clock. The determination front is set by the antagonistic activities of the FGF pathway in the posterior PSM and the retinoic acid (RA) pathway in the anterior PSM (Moreno and Kintner, 2004; Goldbeter et al., 2007). It moves toward the posterior extremity during embryo elongation. A prospective somite consists of the cells left behind the determination front during one oscillation of the clock. Oscillations cease in these cells and the expression of certain genes changes from a cyclic to a stable pattern, restricted to part of the future somite. Furthermore, these cells express new segmentation genes, in either the anterior or the posterior half of the future somites. This contributes to their antero-posterior polarity and prefigures morphological segmentation (Mara and Holley, 2007; Dequéant and Pourquié, 2008; Aulehla and Pourquié, 2010; Gibb et al., 2010).

The players in somite segmentation include components of the Notch, RA, FGF and Wnt signalling pathways, and molecules involved in the post-transcriptional control of gene expression. Indeed, the stability of *Fgf8* mRNA shapes the postero-anterior FGF gradient, but the factors controlling *Fgf8* mRNA degradation remain unknown (Dubrulle and Pourquié, 2004), and the oscillations imply a rapid decay of clock mRNAs (Cibois et al., 2010a). One key post-transcriptional regulator of somite segmentation is Celf1 (previously known as EDEN-BP or Cugbp1), a multifunctional RNA-binding protein (Barreau et al., 2006). The knockdown of *celf1* expression impairs Xenopus segmentation. Celf1 protein interacts directly with *rbpj* mRNA [also known as *suppressor of hairless*, *su(h)*] (Gautier-Courteille et al., 2004), and two lines of arguments indicate that the loss of the interaction between Celf1 protein and *rbpj* mRNA is the main cause of impaired segmentation of *celf1* morphants. First, *rbpj* is overexpressed in Celf1-deficient embryos, consistent with the capacity of Celf1 to target bound mRNAs to rapid deadenylation and decay. Furthermore, *rbpj* overexpression (driven by mRNA injection) is sufficient to strongly alter segmentation (Gautier-Courteille et al., 2004). Second, impairing the interaction between Celf1 protein and *rbpj* mRNA with a “target protector” antisense morpholino yielded a moderate *rbpj* overexpression, both at the mRNA and protein levels, and recapitulated the segmentation defects. A minute amount of a second morpholino blocking *rbpj* translation, which drove Rbpj protein abundance back to its initial level, restored segmentation, strongly supporting the specificity of the target protector morpholino (Cibois et al., 2010b). These experiments have revealed the phenotypic consequences of *rbpj* overexpression for the first time in vertebrates, and have highlighted a post-transcriptional mechanism that prevents *rbpj* overexpression.

*rbpj* encodes a DNA-binding protein that plays a key role in the Notch signalling pathway. Upon binding to its ligand, the Notch transmembrane receptor is cleaved, releasing its intracellular domain (NICD). This is subsequently translocated to the nucleus, where it associates with Rbpj. The Rbpj–NICD complex activates the transcription of target genes, but, in the absence of NICD, Rbpj represses them (Kovall and Blacklow, 2010). Somite segmentation is impaired in both embryos overexpressing *rbpj* (Cibois et al., 2010b) and in *rbpj* mutants and morphants (Oka et al., 1995; Echeverri and Oates, 2007). Segmentation therefore requires the presence of optimal amounts of Rbpj protein. In this study, we investigated the consequences of Rbpj overproduction for the Notch pathway and for other signalling pathways involved in Xenopus segmentation.

## Results

### *rbpj* overexpression modulates Notch signalling in the presomitic mesoderm

**Fig. 1.**
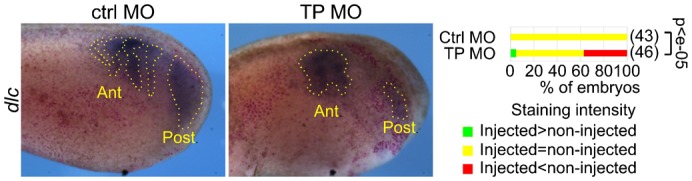
Impact of *rbpj* overexpression on *dlc* expression. We injected nLacZ mRNA and control (left panel) or the target-protector (middle panel) morpholinos into one blastomere of two-cell Xenopus embryos and allowed the embryos to develop to the tailbud stage. We then stained them for β-galactosidase activity (red dots) and *dlc* by *in situ* hybridisation (ISH). The right panel shows the percentage of embryos with a staining intensity in the injected side above, equal to, and below (respectively green, yellow and red) that in the control side. We compared the distributions between these three classes by a chi-square test and we show the p-value. The photographs are lateral views of the injected sides, anterior left. The positions of the anterior PSM (Ant) and the posterior PSM (Post) are indicated.

A target-protector morpholino (TP MO) causes *rbpj* overexpression by abolishing the repressive interaction between Celf1 protein and *rbpj* mRNA (Cibois et al., 2010b). In Drosophila, some phenotypes associated with *rbpj* [*Su(H)*] overexpression are similar to a Notch gain-of-function while other phenotypes are similar to a Notch loss-of-function. This is probably a consequence of the dual capacity of this protein to activate and repress transcription (Furriols and Bray, 2000). Similarly, in Xenopus segmentation, TP MO-mediated *rbpj* overexpression could either enhance or attenuate Notch signalling. To discriminate between these possibilities, we injected the TP MO unilaterally with a lineage tracer and analysed the expression of *dlc* (*delta-2*). *dlc* encodes the main Notch ligand in the PSM. It is expressed in the posterior PSM and as 3–4 chevrons in the anterior PSM. The TP MO repressed the posterior expression of *dlc*, and transformed the stripes in the anterior PSM to a more continuous pattern by filling the gaps (Fig. 1). The expression of *dlc* in the PSM is controlled by complex feedback loops. Stimulating the Notch pathway with a constitutively active mutant of Rbpj repressed *dlc* in the posterior PSM, and to a weaker extent in the anterior PSM. Conversely, repressing the Notch pathway with a dominant negative mutant of Rbpj filled the gaps in the anterior PSM and weakly stimulated its expression in the posterior PSM (Jen et al., 1997; Sparrow et al., 1998; Jen et al., 1999). Hence, the TP MO mimicks the effect on *dlc* of a Notch gain-of-function and a Notch loss-of-function, respectively, in the posterior and the anterior PSM. This differential sensitivity of Notch signalling to Rbpj abundance may reflect the differential amounts of NICD in the two compartments of the PSM, but we were unable to accurately compare the amounts of NICD in these two compartments.

### *rbpj* overexpression represses retinoic acid signalling in the presomitic mesoderm through *cyp26a* upregulation

**Fig. 2.**
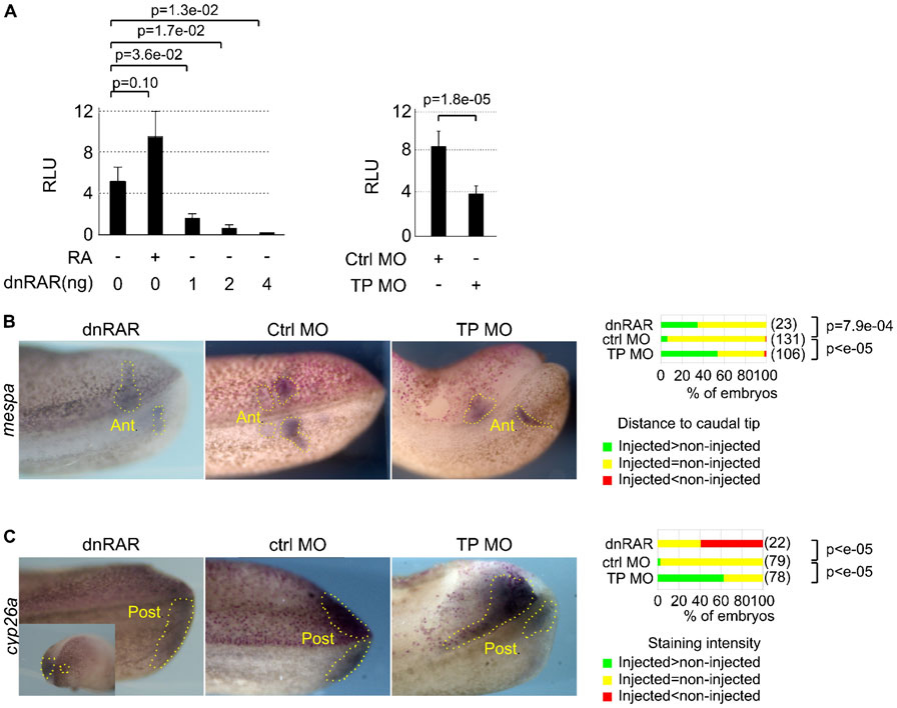
Impact of *rbpj* overexpression on the RA signalling pathways. (**A**) We injected the pRARE-luciferase plasmid and dnRAR mRNA or control or TP morpholinos together into one blastomere of two-cell embryos, which we allowed to develop to the early tailbud stage. We treated the embryos with retinoic acid where indicated, and we extracted proteins and measured luciferase activities (relative light unit). We show here the mean luciferase activities, ± SEM, of 8–16 embryos per condition. We compared the mean values between the controls and the other conditions by a Student's t-test and we show the p-values. (**B**) We injected nLacZ mRNA and dnRAR or control or the target-protector morpholinos, we allowed the embryos to develop to the tailbud stage and we stained them for β-galactosidase activity and *mespa* mRNA as in Fig. 1. We compared the distances between the posterior tip of the embryo and the posterior limit of *mespa* expression, and the right panel shows the percentage of embryos with a distance in the injected side above, equal to, and below (respectively green, yellow and red) that in the control side. We compared the distributions, between these three classes, of the control and the other conditions by a chi-square test and we show the p-values. The photographs are dorsal views, anterior left, injected-side up. Panel C is the same as panel B, but the ISH revealed *cyp26a* expression. The right panels show the percentage of embryos with a staining intensity in the injected side above, equal to, and below (respectively green, yellow and red) that in the control side. The photographs are dorsal views, anterior left, injected-side up, except the posterior views.

We next analysed the effects of the TP MO on the retinoic acid (RA) signalling pathway. We injected a plasmid carrying the luciferase reporter under the control of RA response elements into embryos (Blumberg et al., 1997). The addition of exogenous RA stimulated the luciferase activity, whereas the dominant negative RA receptor dnRAR repressed it (Fig. 2A). This indicates that luciferase activity adequately reflects RA signalling. Importantly, the TP MO lowered the luciferase activity (Fig. 2A), suggesting that *rbpj* upregulation represses the RA signalling pathway.

To confirm these data, we analysed the expression of *mespa* (*mesoderm posterior homologue A*, *thylacine-1*, *Mesp2* in mice). This gene marks the determination front. Because the position of the front is contributed by an antero-posterior gradient of RA signalling, stimulating the RA pathway shifts posteriorly its expression while repressing the RA pathway shifts it anteriorly (Moreno and Kintner, 2004; Oginuma et al., 2008). The TP MO shifted anteriorly *mespa* domain of expression, pretty much like dnRAR-mediated inhibition of RA signalling (Fig. 2B). This confirms that the RA pathway is repressed in embryos overexpressing *rbpj*.

Another gene that positively responds to RA signalling is *cyp26a* (Moreno and Kintner, 2004). However, we found that the TP MO, unlike dnRAR, stimulated *cyp26a* (Fig. 2C). It is worth noting that *cyp26a* is overexpressed in the posterior PSM, at the place where the TP MO stimulates the Notch pathway. We think that the upregulation of *cyp26a* is the main reason of RA attenuation in TP MO-injected embryos for three reasons. First, *cyp26a* encodes an RA-degrading enzyme. Second, in zebrafish, the morpholino-mediated knockdown of *rbpj* repressed *cyp26a* expression, whereas a dominant activated mutant of *rbpj* activated it (Echeverri and Oates, 2007). Third, if Cyp26a did not link the stimulation of Notch to the repression of RA signalling, then *cyp26a* would only be a downstream target of RA signalling, and we would have observed a repression, rather than a stimulation, of *cyp26a* expression in TP MO-injected embryos. We conclude that overexpressed *rbpj* stimulates *cyp26a*, which represses the RA pathway.

### *rbpj* overexpression represses FGF signalling in the presomitic mesoderm

**Fig. 3.**
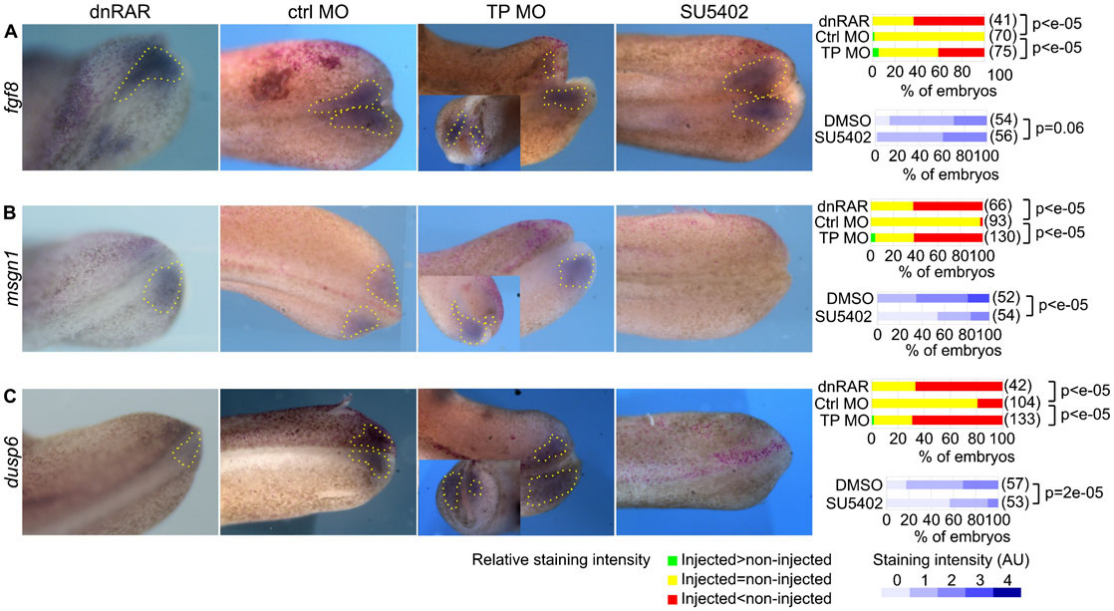
Impact of *rbpj* overexpression on the FGF signalling pathways. We injected nLacZ and control or TP MO, or dnRAR mRNA, as indicated, into one blastomere of two-cell embryos, which we allowed to develop until the tailbud stage. Where indicated, we treated the embryos for 2 hours with SU5402 (embryos injected with control MO were treated with DMSO) and we stained them for β-galactosidase activity (red dots) and *fgf8* (**A**), *msgn1* (**B**), or *dusp6* (**C**) mRNA by ISH. We sorted DMSO and SU5402-treated embryos into 5 classes depending on their staining intensities. The right panels show the percentages of embryos within each of these classes, and, for injected embryos, the percentage of embryos with a staining intensity in the injected side above, equal to, and below (respectively green, yellow and red) that in the control side. We compared the distributions, between the classes, of the control and the other conditions by a chi-square test and we show the p-values. All the photographs are dorsal views, anterior left, injected-side up.

We next investigated the effects of *rbpj* overexpression on the FGF pathway. The TP MO repressed *fgf8* expression (Fig. 3A). Because *rbpj* overexpression attenuates RA signalling (see above), and dnRAR repressed *fgf8* (Fig. 3A) as previously described (Moreno and Kintner, 2004), the repression of *fgf8* in TP MO-injected embryos may be due to RA signalling inhibition. *fgf8* encodes the relevant FGF ligand in the PSM (Dubrulle et al., 2001), and the downregulation of *fgf8* expression was expected to translate into attenuation of FGF signalling. We checked this point by analysing the expression of *msgn1* (*mesogenin-1*, *mespo*) and *dusp6* (*mkp3*). The FGF pathway controls these two genes, in conjunction with the Wnt [*msgn1* (Wang et al., 2007)], or the RA [*dusp6* (Moreno and Kintner, 2004)] pathways. Accordingly, the FGF pathway inhibitor SU5402 repressed these two genes (Fig. 3B,C). These two genes were also downregulated in TP MO-injected embryos (Fig. 3B,C), and this supports our interpretation that *rbpj* overexpression represses FGF signalling.

### *rbpj* overexpression takes over FGF inhibition in the control of the RA pathway

**Fig. 4.**
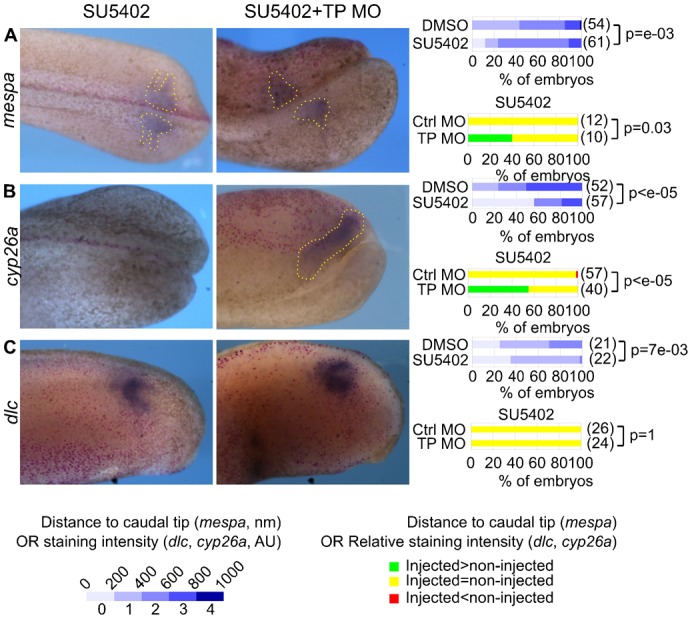
Rbpj mediates the repression exerted by FGF signalling on the RA pathway. We injected nLacZ mRNA and control (left panel) or the TP (middle panel) morpholinos into one blastomere of two-cell embryos and allowed them to develop to the tailbud stage. We treated embryos for 2 hours with SU5402. We detected β-galactosidase activity and *mespa* (**A**), *cyp26a* (**B**) or *dlc* (**C**) mRNAs by Red-gal staining and ISH. We also treated control morpholino-injected embryos with DMSO, stained them with the same probes, and we sorted DMSO and SU5402-treated embryos into 5 classes depending on their staining intensities (*dlc*, *cyp26a*) or the distance between the posterior tip of the embryo and the posterior limit of expression (*mespa*), and the right panels show the percentages of embryos within each of these classes. For injected embryos treated with SU5402, the right panels also shows the percentage of embryos with a staining intensity or a distance to posterior tip in the injected side above, equal to, and below (respectively green, yellow and red) that in the control side. We compared the distributions, between the classes, of the control and the other conditions by a chi-square test and we show the p-values. (A,B) dorsal views, anterior left, injected-side up, (C) lateral views of the injected side, anterior left.

Moreno and Kintner showed that the FGF and RA pathways were mutually antagonistic, so that FGF repression was associated with enhanced RA signalling (Moreno and Kintner, 2004). However, our results demonstrate that *rbpj* upregulation represses both RA and FGF signalling. To solve this apparent discrepancy, we analysed embryos injected with the TP MO and exposed to SU5402. While SU5402 shifted posteriorly the expression of *mespa*, consistent with an RA gain-of-function, the TP MO reversed this effect (Fig. 4A). Similarly, SU5402 repressed *cyp26a*, but the TP MO restored a high level of expression of *cyp26a* in SU5402-treated embryos (Fig. 4B). Consequently, SU5402 and the TP MO have opposite consequences on *mespa* and *cyp26a* expression, but the consequences of exerting these two treatments simultaneously are similar to the consequences of TP MO injection. By contrast, the TP MO had no effect on *dlc* in SU5402-challenged embryos (Fig. 4C). Together, these data show that *rbpj* overexpression takes over FGF repression in the control of RA signalling, as deduced from *cyp26a* and *mespa* expression, but not in the control of *dlc* expression.

## Discussion

Pioneering work in Xenopus revealed that *cyp26a* encodes an RA-degrading enzyme and is positively controlled by the FGF pathway, while *dusp6* encodes a phosphatase that antagonizes FGF signalling and is positively controlled by RA. This results in a mutual antagonism between the RA and FGF pathways that defines two compartments of the PSM, a posterior one where FGF predominates and an anterior one where RA predominates (Moreno and Kintner, 2004). The present article extends this work in two directions.

**Fig. 5.**
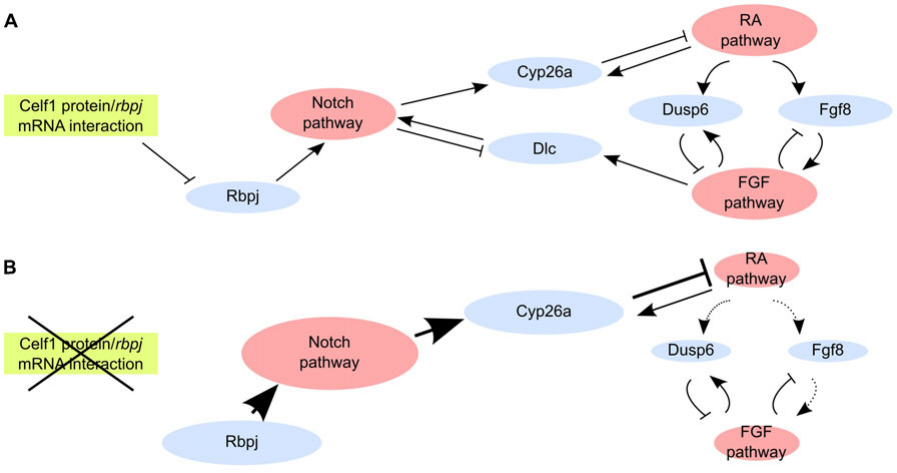
Model for the relationships between the RA, FGF and Notch pathway in Xenopus PSM. Three intra-pathway feedback loops attenuate each pathway (RA stimulate *cyp26a* and FGF stimulates *dusp6* while Notch represses *dlc*). In addition, the RA pathway either stimulates (through *fgf8*) or represses (through *dusp6*) the FGF pathway, the FGF pathway stimulates the Notch pathway (through *dlc*), and the Notch pathway represses the RA pathway (through *cyp26a*). (**A**) The Celf1 RNA-binding protein attenuates the Notch pathway by limiting the abundance of Rbpj protein through a post-transcriptional control. Notch signalling depends on FGF signalling through Dlc. (**B**) When the TP MO impairs the repressive interaction between Celf1 and *rbpj* mRNA, *rbpj* is overexpressed, which stimulates the Notch pathway. Consequently, the RA pathway is repressed (due to *cyp26a* overexpression) and the FGF pathway is repressed (due to *fgf8* repression). FGF repression does not lead to Notch repression owing to *rbpj* overexpression, and relieving the repressive interaction between Celf1 protein and *rbpj* mRNA uncouples the Notch and RA pathways from the FGF pathway.

First, we observed that inhibiting the RA pathway either directly (by injecting dnRAR mRNA) or indirectly (by injecting the TP MO) repressed *msgn1*, probably owing to *fgf8* repression (Fig. 3). Conversely, it had previously been reported that dnRAR activated *msgn1* expression owing to *dusp6* repression (Moreno and Kintner, 2004). A plausible explanation for this discrepancy is the age of the embryos used. Indeed, we examined the effects of dnRAR on *msgn1* expression in tailbuds, whereas neurulae were previously analyzed. Nevertheless, taking *msgn1* expression as a proxy for FGF signalling, our results suggest that the relationships between the RA and FGF pathways can not be only summarized as a mutual antagonism but that, at least under certain circumstances, RA signalling is able to positively control the FGF pathway (Fig. 5). Cell migration is a major contributor to embryo elongation in fish (Zhang et al., 2008) and, in chick, FGF signalling in the PSM directs cell motions leading to the antero-posterior elongation of the embryo (Bénazéraf et al., 2010). The dependence of posterior FGF signalling on anteriorly produced RA may thus control embryo elongation rate. It is therefore tempting to attribute embryo curvature (e.g. Fig. 2B, Fig. 4A) to a decreased elongation of the TP MO-injected side, due to the repression of RA and consequently FGF signalling.

Second, our results prompt us to reconsider the relationships between the FGF, Notch and RA pathways in segmentation. TP MO-mediated overexpression of *rbpj* repressed *dlc* and stimulated *cyp26a*. Because the Notch pathway represses *dlc* (Jen et al., 1997; Sparrow et al., 1998; Jen et al., 1999) and *cyp26a* is under Rbpj control (Echeverri and Oates, 2007), these observations indicate that *rbpj* overexpression stimulates Notch signalling in the posterior PSM. TP MO-mediated stimulation of Notch signalling repressed RA signalling, while attenuating the FGF pathway by SU5402 treatment stimulated the RA pathway. Importantly, simultaneously activating the Notch pathway and repressing the FGF pathway led to RA repression, as deduced from *cyp26a* overexpression and *mespa* anterior shift. This shows that *rbpj* overexpression takes over FGF repression in the control of RA signalling. Furthermore, FGF may positively control the Notch pathway, since FGF repression downregulated *dlc*. This suggests that the Notch pathway is an intermediate by which the FGF pathway represses RA signalling. A demonstration of this model would be to show that Rbpj is required for the repression of RA induced by FGF signals. Unfortunately, we were unable to make FGF gain-of-function experiments, because premature FGF activation strongly alters gastrulation. Taking this caveat into account, we propose that, in a control situation, Celf1 minimises Rbpj protein abundance to keep Notch signalling at a level compatible with FGF regulation, ensuring a coupling between the FGF and RA pathways (Fig. 5A). When the repression of *rbpj* by Celf1 is abolished by the TP MO, *rbpj* overexpression leads to high, FGF-independent Notch signalling that results in *cyp26a* overexpression and RA attenuation (Fig. 5B). Hence, the post-transcriptional control exerted by Celf1 protein on *rbpj* mRNA is required to couple the FGF with the Notch and RA pathways in somite segmentation.

The Notch pathway plays a central role in the clock (Mara and Holley, 2007; Dequéant and Pourquié, 2008; Aulehla and Pourquié, 2010; Gibb et al., 2010), and we propose here that it also contributes to the cross-talk between the FGF and RA pathways to determine the determination front. Somite segmentation is impaired in *rbpj* mutants and morphants (Oka et al., 1995; Echeverri and Oates, 2007), but also in embryos overexpressing *rbpj* (Cibois et al., 2010b). Segmentation therefore requires the presence of optimal amounts of Rbpj protein. This requirement for such a tight control of Rbpj abundance was not expected given the ubiquitous expression of the *rbpj* gene (Wettstein et al., 1997). *celf1* is also required for zebrafish segmentation (Matsui et al., 2012) and is expressed in mice PSM (Kress et al., 2007), and it will be important to test if the function of Celf1 to control *rbpj* is conserved in the segmentation process of other vertebrates.

## Materials and Methods

### Plasmids and probes

The plasmids encoding *dlc*, nuclear β-galactosidase (nLacZ, pCS2+nBGal vector) and dnRAR have been described (Blumberg et al., 1997; Gautier-Courteille et al., 2004). *mespa* ORF was PCR-amplified using forward (ATGGATTTCTCTCCAACAAAAC) and reverse (TTAATAAGCAAGATGCTGAAGTG) primers and Xenopus embryo cDNA, and cloned in pGEM-T (Promega). Other plasmids were purchased from Imagene (*fgf8*, IMAGp998E1914583Q; *msgn1*, IRAKp961C13163Q; *dusp6*, IRBHp990F0612D; *cyp26a*, IRBHp990D0634D). Antisense probes for ISH and sense mRNAs were obtained from the above plasmids by in vitro transcription (Promega Riboprobe kit with Boehringer dig-UTP and Ambion Megascript kit respectively).

### Xenopus laevis embryos procedures

We injected embryos at the two-cell stage in one blastomere with one or several of the following: 2 pmol of control or target-protector morpholino (Cibois et al., 2010b), 0.5 fmol of nLacZ mRNA, 5 fmol of dnRAR RNA, 1.5 fmol of hGR-NICD or hGR-Su(H)-Ank mRNA. They were allowed to develop at 16–22°C. When required, tailbud (stage 24–26) embryos were incubated for 2 hours at 22°C with RA (1 µM, Sigma), cycloheximide (10 µg/ml, Sigma), SU5402 (400 µM), dexamethasone (20 µM), or a concentration of DMSO or ethanol corresponding to the highest amount in the drug solutions. For ISH, embryos were fixed 45 min in MEMFA, rinsed in PBS and preincubated in PBS supplemented with 2 mM MgCl_2_. They were revealed for β-galactosidase activity by incubation in X-Gal mixer (35 mM K_3_Fe(CN)_6_, 35 mM K_4_Fe(CN)_6_, 2 mM MgCl_2_ in PBS) with 1 mg/ml Red-Gal (Sigma) and refixed for 1 h in MEMFA. ISH were as described (Harland, 1991). We measured luciferase activity with the Promega luciferase assay system after crushing individual embryos in 100 µl PLB (Promega).

### Scoring and statistical analyses

For *mespa* we used a grid to measure the distance between the posterior tip of the embryo and the posterior limit of expression. For the other probes, staining was quantified by scoring blindly the injected side of each embryo on a scale from 0 (no expression) to 4 (very strong expression). Alternatively, we compared the injected and non-injected sides of the embryos and sorted the embryos according to the relative expression levels in the two sides. We made the statistical analyses with the R software. For the chi-square test, the p-values were calculated by Monte Carlo simulation with 100,000 replicates. Hence, p-values below e-05 could not be precisely measured.
